# Correlating novel variable and conserved motifs in the Hemagglutinin protein with significant biological functions

**DOI:** 10.1186/1743-422X-5-91

**Published:** 2008-08-05

**Authors:** Deena MA Gendoo, Mahmoud M El-Hefnawi, Mark Werner, Rania Siam

**Affiliations:** 1 YJ-Science and Technology Research Center (STRC), American University in Cairo, Cairo, Egypt; 2 Department of Biology, American University in Cairo, Cairo, Egypt; 3 Department of Informatics and Systems, Division of Engineering Sciences Research, National Research Centre (NRC), Cairo, Egypt; 4 Department of Mathematics and Actuarial Science, American University in Cairo, Cairo, Egypt

## Abstract

**Background:**

Variations in the influenza Hemagglutinin protein contributes to antigenic drift resulting in decreased efficiency of seasonal influenza vaccines and escape from host immune response. We performed an in silico study to determine characteristics of novel variable and conserved motifs in the Hemagglutinin protein from previously reported H3N2 strains isolated from Hong Kong from 1968–1999 to predict viral motifs involved in significant biological functions.

**Results:**

14 MEME blocks were generated and comparative analysis of the MEME blocks identified blocks 1, 2, 3 and 7 to correlate with several biological functions. Analysis of the different Hemagglutinin sequences elucidated that the single block 7 has the highest frequency of amino acid substitution and the highest number of co-mutating pairs. MEME 2 showed intermediate variability and MEME 1 was the most conserved. Interestingly, MEME blocks 2 and 7 had the highest incidence of potential post-translational modifications sites including phosphorylation sites, ASN glycosylation motifs and N-myristylation sites. Similarly, these 2 blocks overlap with previously identified antigenic sites and receptor binding sites.

**Conclusion:**

Our study identifies motifs in the Hemagglutinin protein with different amino acid substitution frequencies over a 31 years period, and derives relevant functional characteristics by correlation of these motifs with potential post-translational modifications sites, antigenic and receptor binding sites.

## Background

Molecular and viral characterization of the hemagglutinin protein (HA) from different hosts has increased in the last three decades, in response to three worldwide outbreaks of influenza in the years 1918, 1957, and 1968 [1]. The H3N2 antigenic subtype responsible for the 1968 pandemic was first isolated in July 1968 in Hong Kong, and supplanted the H2N2 virus responsible for the 1957 Asian flu pandemic[2,1].

Bioinformatics and computational approaches towards molecular understanding of HA have largely focused on the determination of mutation levels and evolution of the HA gene, and identification and prediction of antigenic variants of H3N2 by locating potential immunodominant positions on the HA protein. Phylogenetic analysis of H3N2 genomes illustrates that the H3N2 virus is composed of multiple and distinct clades, which exhibit genetic variation by interacting with minor lineages through reassortment events [2]. Whole-genome alignments, statistical analysis with construction of evolutionary trees were used to identify locations of mutations within H3N2, predict their yearly frequency, and determine modes of antigenic drift and positive selection [2]. Using a parsimonious tree to map the HA1 domain of 254 H3N2 viral genes, Fitch and coworkers determined that HA1 evolves at an average rate of 5.7 nucleotide substitutions/year, and indicated the presence of six hypervariable codons of the HA gene which accumulate replacement substitutions at a rate that is 7.2 times that of other codons [3]. Some studies have concluded that H3 hemagglutinin gene exhibits positive selection in key regions of the HA molecule such as the receptor-binding site and antibody-binding sites [4], which result in new antigenic and resistant strains. Several studies used bioinformatics approach to predict antigenic strains of the H3N2 virus [5-7]. One study generated a model based on 131 positions in the five antigenic sites of the protein, and which could predict antigenic variants of H3N2 with an agreement rate of 83% to existing serological data [5]. Later studies also identified twenty amino acids positions, which are potential immunodominant positions and contribute to antigenic difference between strains [6].

To the best of our knowledge, few bioinformatics publications have addressed motif search in segments of the H3N2 genome where mutations have been observed. A recent study by Ahn and Son [7] aimed to detect relative synonymous codon usage (RSCU) and codon usage patterns (CUP) in HA and Neuraminidase (NA) from H3N2, H9N2, and H5N1 subtypes within human, avian, and swine populations. They established a unique CUP for each subtype, and observed a possible divergence within human H3N2 isolates based on their synonymous CUPs. A study published earlier this year [8] has focused specifically on the H3N2 subtype, using nucleotide co-occurrence networks of human H3N2 strains to predict H3N2 evolution. However, analysis of H3N2 nucleotide and protein genomes to discover patterns and motifs yet remains to be elucidated. In this study, we report motifs and assign potential functional characteristics within the HA protein sequences of the gene of H3N2 human influenza isolates from Hong Kong between 1968 and 1999. We identify motifs within the HA protein, and interrelate these motifs with amino acid substitutions frequency, co-mutating pairs, potential post-translation modification sites, antigenic sites, receptor-binding sites. We focus our analysis on motifs with varying mutation frequency and correlate the variable motif with a high number of potential post-translational modification sites that overlap antigenic and receptor binding sites. We speculate that mutation in these motifs results in the emergence of viral strains that are highly pathogenic and has the intrinsic character to overcome that host defense mechanisms.

## Results

### 14 MEME Blocks identified from HA1 consensus sequences; representatives of strains isolated from 1968 to 1999

**Figure 1:**
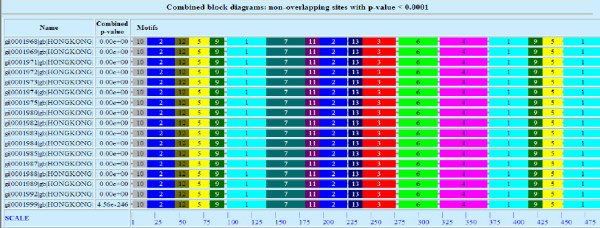
**Selected 14 MEME Blocks in the HA1 consensus sequence from 1968–1999**. Combined block diagram of non overlapping sites with p value < 0.0001 was generated from the MEME server which are common to the entire data set, with the exception of block 14 which occurs in only 16 of the 17 sequences.

Submission of the 17 HA1 consensus sequences generated from the nucleotide GenBank accession numbers (refer to the material and methods section) to the MEME server has generated 50 protein motifs from which we selected 14 MEME blocks which are common to the entire data set (Figure 1), with the exception of block 14 which occurs in only 16 of the 17 sequences. All the observed blocks had a p value < 0.0001. MEME blocks 1 and 2 occur 3 times over the entire protein sequence with a motif size of 41 and 29 amino acids respectively. MEME blocks 3, 5, 9 and 10 occur twice over the entire amino acid sequence with a motif size of 35, 21, 15 and 11 respectively. The remaining MEME blocks occur only once with varying motif sizes of 4–50 amino acids. Table 1 shows the location of each block within the HA sequence. Notably, all of the blocks occur at least once within the HA1 domain (17–344) with the exception of blocks 8 and 14, which only occurs in HA2.

**Table 1:** MEME blocks positions, size and genetic distance

**MEME BLOCK**	**Start Position**	**End Position**	**Block Size (amino acids)**
MEME 1	89	129	41
	348	388	41
	426	466	41
MEME 2	14	42	29
	179	207	29
	478	506	29
MEME 3	507	541	35
	215	249	35
MEME 4	296	345	50
MEME 5	404	424	21
	49	69	21
MEME 6	253	293	41
MEME 7	130	170	41
MEME 8	542	562	21
MEME 9	71	85	15
	389	403	15
MEME 10	3	13	11
	467	477	11
MEME 11	171	178	8
MEME 12	43	48	6
MEME 13	209	214	6
MEME 14	563	566	4

HA consensus sequences were submitted in Multiple Em for Motif Elucidation (MEME) server. The fourteen MEME blocks spanning the consensus sequence alignment are presented, with the start and end positions and width of each block.

### Genetic distance and entropy analysis of MEME blocks reveals variable and conserved motifs

**Figure 2:**
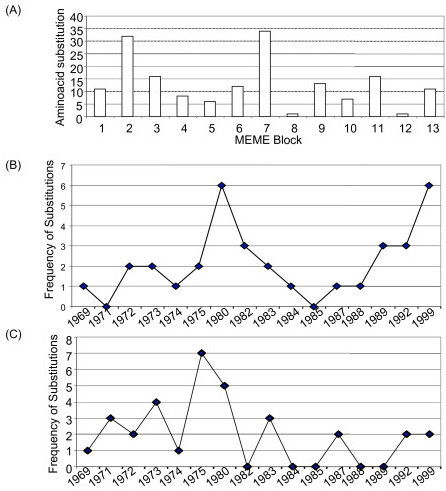
**Number of aminoacid substitutions in each MEME block over the period from 1968–1999**. (A) Bar graph of amino acid substitutions within MEME blocks for each of the years. (B) Behavior of the substitutions in MEME block 7; frequency of amino acid substitutions within MEME block 7 largely follows the occurrence pattern of substitutions within the entire protein as illustrated Table 2, reaching a peak in 1980, which corresponds to the year with the greatest number of mutations in the alignment. (C) Behavior of the substitutions in MEME block 2.

Amino acid substitutions over the 1968–1999 data set were extracted from the multiple sequence alignment using MEGA 4.0 [9]. The numbers of amino acid substitutions in the 17 consensus sequence were determined by Infoalign and are tabulated in Table 2. We compared the percent change in amino acid substitution (mutation frequency) in the Hong Kong data set from 1968–1999 and calculated the genetic distance. Two of the years, investigated in our study, showed significant amino acid substitutions; in 1975 fifteen amino acid substitutions are observed with a 2.65 percent change from 1974 and in 1983 thirteen amino acid substitutions are observed with ~2.3 percent change from 1982 (Table 2). Association between amino acids substitution and the MEME blocks were determined and are represented in Figure 2a. We subdivided the blocks into 3 categories based on the genetic distance (Figure 2a); highly variable motifs include MEME blocks 7, 11, and 13, highly conserved motifs include blocks 1 and 8, and the rest of the MEME motifs showed intermediate variability (Table 2).

**Table 2:** Amino acid substitutions in the different isolates from 1969–1999 used to extrapolate the genetic distance in the different MEME blocks

**YEARS**	**Number of** **amino acid** **substitutions**	**% CHANGE** **BETWEEN YEARS**	**MEME** **Block**	**Genetic** **Distance**
1968–1969	5	0.883392	**1**	**0.08943**
1969–1971	12	1.943463	2	0.3678
1971–1972	11	1.943463	3	0.228
1972–1973	22	3.886926	4	0.16
1973–1974	5	0.883392	5	0.142
1974–1975	15	2.650177	6	0.293
1975–1980	29	4.946997	*7*	*0.8292*
1980–1982	6	1.060071	**8**	**0.048**
1982–1983	13	2.296820	9	0.288
1983–1984	2	0.353357	10	0.212
1984–1985	1	0.176678	*11*	*2*
1985–1987	7	1.236749	12	0.167
1987–1988	3	0.530035	*13*	*1.833*
1988–1989	8	1.423488		
1989–1992	10	2.473498		
1992–1999	23	4.240283		

Using ClustalW alignment the number of observed substitutions for each of the consensus sequence and the equivalent years are tabulated using Infoalign. The highest aminoacid substitution (29 aa substitutions over the entire sequence) was in Years 1980. The genetic distance in each MEME block is calculated showing that MEME blocks 1 and 8 are conserved (bold), MEME blocks 7, 11 and 13 are highly variable and the other MEME blocks show intermediate variability.

In an attempt to establish the relationship between blocks and amino acids substitutions over the time period between 1968–1999, a line graph was drawn to examine the mutation rate of each of the MEME blocks, in order to infer the evolutionary behavior of the motifs (i.e. whether they were acted upon by positive selection or neutral genetic drift evolution). The frequency of amino acid substitutions within the highly variable MEME block 7 (Figure 2b) largely follows the occurrence pattern of substitutions within the entire protein (Table 2), reaching a peak in 1980, which corresponds to a year with a high number of mutations in the alignment, and following a similar zenith in 1985. However, for the intermediately variable MEME block 2, not all the mutations within each year of the alignment occur in the block, resulting in a zigzag behavior from 1982 onwards (Figure 2c). Some blocks only undergo amino acids substitutions in one or two years of the cohort, as is the case with motifs 8 and 12 (data not shown). MEME block 5 undergo amino acids substitutions from 1968 to 1984, then remain conserved after this period (data not shown). Similarly, MEME block 10 is conserved after 1984 with an exception of an amino acid substitution in 1992 (data not shown). Additionally, certain blocks remain conserved for a few years of the cohort, but undergo amino acids substitutions towards the later years of the study. Notable examples include blocks 6, 9, and 13 (data not shown). The MEME program lists the HA MEME blocks in descending order based on their e-value, as such, MEME blocks 2 and 7 are quite significant and plausible for further analysis.

**Figure 3:**
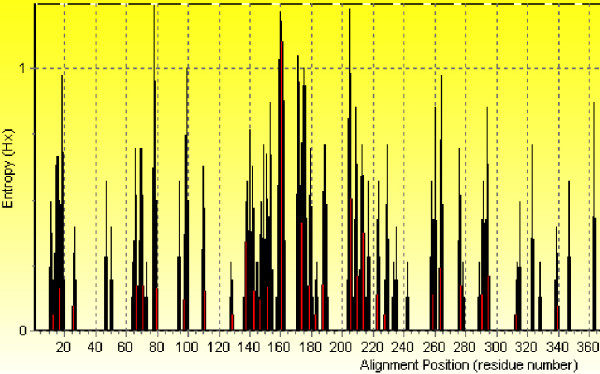
**Entropy plot of the protein consensus ClustalW alignment**. Amino acid positions that do not exhibit any changes over the years have entropy of 0, whereas positions of high variability are represented by peak in the plot. Two hot spots of variability were observed and are clustered around amino acid position 140–190, and 200–240. The entropy analysis was performed for the entire hemagglutinin sequence (560 amino acids), but at amino acid position 340 (HA2) the analysis does not exhibit much entropy.

To confirm these finding, we correlated hot spots of variability with MEME blocks, using an entropy plot of the HA alignment (Figure 3). Hot spots of variability are clustered around amino acid position 140–190, and 200–240. Through out this study, we define a hot spot cluster as a 40 amino acid block containing more than 35% of amino acid substitutions. The first part of hot spot cluster I between amino acid position 140–154, is included within MEME block 7 (130–170). The second part of hot spot cluster I, between position 170–180, overlaps MEME block 11 entirely (171–178) and with one of the repetitive MEME block 2 (179–207). Hot spot cluster II overlaps entirely MEME block 13 (209–214) and almost entirely MEME block 3 (215–249). The two significant hot spots of variability were confirmed by looking at conserved regions generated by BIOEDIT, with a minimum length of 15 amino acids and maximum entropy 0.2, and this region did not overlap with the conserved region analysis (data not shown).

### Potential post-translational modification sites in HA protein

Scanning the 17 consensus sequence against the existing Prosite Motifs database (PPSearch) revealed five potential post-translational modification sites. The sites detected include 24 phosphorylation, 12 glycosylation and 14 myristylation sites (Table 3). 7 of the potential phosphorylation sites are Casein kinase II (CKII) phosphorylation sites encompassing different region of the protein. One study has previously reported a CKII phosphorylation domain [10]. 16 of the potential phosphorylation sites are Protein kinase C (PKC) phosphorylation site encompassing different regions of the protein. The clustering of the PKC phosphorylation site is at position 152–224 (9/16 sites are in this region) in contrast to the clustering of CKII phosphorylation site from position 416–459); it is worth noting that CKII phosphorylation clustering is followed by two PKC phosphorylation sites. One cAMP- and cGMP-dependent protein kinase phosphorylation site was identified at position 156–159 (within the single MEME block 7).

**Table 3:** Positions of potential post-translational modification sites

Motif ID	Expression	Start position of the motif	End position of the motif	Years observed
CK2_PHOSPHO_SITE Casein kinase II phosphorylation site.	[ST]-x(2)-[DE].	44	47	1968,1969,1971,1972
		81	84	
		142	145	1972
		203	206	
		416	419	
		432	435	
		456	459	
PKC_PHOSPHO_SITE Protein kinase C phosphorylation site	[ST]-x-[RK]	64	66	All years except 1982
		123	125	
		152	154	
		154	156	
		159	161	
		173	175	1972
		190	192	1975
		203	205	1975, 1980, 1982, 1983, 1984, 1985, 1987, 1988, 1989, 1992
		215	217	
		221	223	
		222	224	
		243	245	
		278	280	
		329	331	
		467	469	
		496	498	
cAMP_PHOSPHO_SITE cAMP- and cGMP-dependent protein kinase phosphorylation site.	[RK](2)-x-[ST]	156	159	1975, 1980, 1982, 1983, 1984, 1985, 1987, 1988, 1989, 1992, 1999
ASN_GLYCOSYLATION N-glycosylation site	N-{P}-[ST]-{P}	24	27	All years except 1971, 1972
		38	41	
		54	57	
		79	82	1975, 1980, 1982, 1983, 1984, 1985, 1987, 1988, 1989, 1992, 1999
		97	100	1968, 1969, 1971, 1972, 1973
		138	141	1999
		142	145	1974, 1980, 1982, 1983, 1984, 1985, 1987, 1988, 1989, 1992, 1999
		149	152	1999
		181	184	
		262	265	1980, 1982, 1983, 1984, 1985, 1987, 1988, 1989, 1992, 1999
		301	304	
		499	502	
MYRISTYL N-myristylation site	G-{EDRKHPFYW}- x(2)-[STAGCN]-{P}.	21	26	
		77	82	1968,1969,1971,1972, 1973, 1974, 1975
		145	150	All years except 1972
		150	155	All years except 1989, 1992
		151	156	All years except 1989, 1992, and 1999
		158	163	1975, 1980, 1982, 1983, 1984, 1985, 1987, 1988, 1989, 1992,
		291	296	1974, 1975, 1980, 1982, 1983, 1984, 1985, 1987, 1988, 1989, 1992,
		302	307	
		346	351	
		349	354	
		361	366	
		376	381	
		495	500	1973, 1974, 1975, 1980, 1982, 1983, 1984, 1985, 1987, 1988, 1989, 1992,
		558	563	All years except 1989,

Prosite motifs detected for the H3N2 sequences using PPSearch this includes 24 phosphorylation, 12 glycosylation and 14 myristylation sites. Potential phosphorylation sites include casein kinase II phosphorylation site, protein kinase C phosphorylation site and cAMP- and cGMP-dependent protein kinase phosphorylation site, ASN glycosylation motifs and N-myristylation sites. The start and end positions of each motif are shown, as well as the regular expression of the motif. Unless otherwise indicated, sites have been observed in all 17 consensus sequences.

Of the 12 ASN glycosylation sites found under PPSearch 7 ASN glycosylation sites have been have been cross-referenced to potential sites of HA in the Uniprot KnowledgeBase, UniProtKB/Swiss-Prot Entry Q91MA7. Of these 7 ASN glycosylation, 5 remain conserved in all years of the data set. Interestingly, 4 ASN glycosylation sites noted by Skehel and co-workers [11] overlap our 2 prominent MEME blocks; 4 ASN glycosylation sites (amino acids 24–27, 38–41, 181–184 and 499–503) overlaps MEME 2 block, and 3 overlaps MEME 7 block (amino acids 138–141, 142–148 and 149–152).

Additionally, 9 of the 14 N-myristylation sites are in MEME blocks 1, 2 and 7. Four sites overlap with MEME block 7, three sites with MEME block1, and two sites with MEME block 2. Interestingly, some of these post-translational modification sites are conserved over the years as is the case with the majority of the phosphorylation sites (>70%), while more than 50% of the glycosylation and myristylation sites are observed in selected years (Table 3). Experimental studies need to be performed to confirm these potential post-translational modification sites.

### Relationship between post-translational modification sites, MEME blocks, amino acid substitutions and entropy

**Figure 4:**
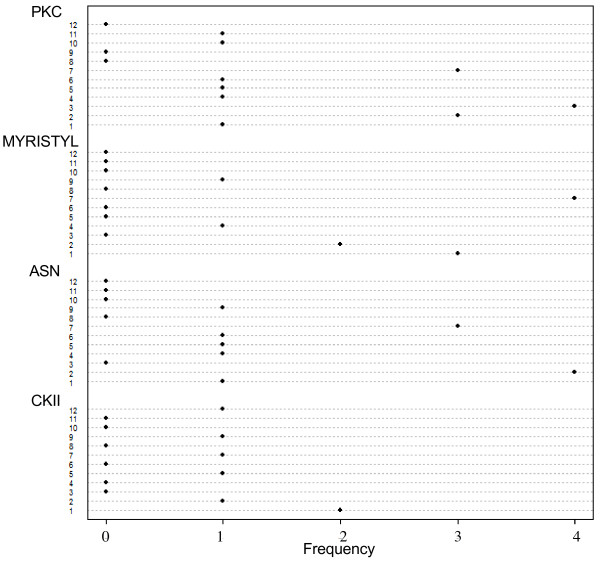
**Frequency of specific potential post-translational modification (prosite) motifs implicated in each of the MEME blocks**. MEME block 7 has the highest number of post-translational modification sites, followed by MEME block 2, 1 and 3 respectively. High frequency of post-translational modification site was recorded when a frequency of 2 or above is observed. Frequency of potential protein kinase C phosphorylation site (PKC) in the MEME blocks reveals that MEME block 3, 2 and 7 have a high PKC sites frequency. Frequency of potential N-myristilation site in the MEME blocks reveals that MEME blocks 1, 2 and 7 have a high myristilation sites frequency. Frequency of potential N-glycosylation site in the MEME blocks reveal that MEME block 2 and 7 has a high glycosylation sites frequency. Frequency of potential CKII phosphorylation sites in the MEME blocks reveals that MEME block 1 and 2 have a high CKII sites frequency.

It was observed that MEME block 7, 2 and 1 contain the greatest number of post-translational modification sites (Prosite motifs) (Figure 4). It is worth noting that only one cAMP-dependent protein kinase phosphorylation site was observed in the dataset, within MEME block 7 and its frequency is therefore not tabulated. An analysis of other post-translational modification sites shows that PKC sites occur mainly within Blocks 2, 3 and 7 while most of the ASN glycosylation sites appear within block 2 and 7 and most myristylation sites appear in MEME block 7 (Figure 4).

**Figure 5:**
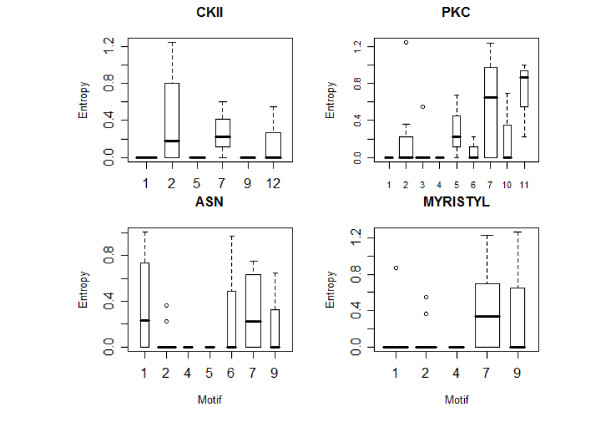
**Average entropy of specific post-translational modification sites in each of the MEME blocks is demonstrated using boxplot**. (A) Average entropy of potential CKII phosphorylation sites in the MEME blocks. Blocks 1, 5 and 9 have zero entropy at all CKII sites. The majority of MEME blocks 2 and 7 CKII sites have nonzero entropy. One of the MEME block 2 CKII sites (amino acid 205) has the largest entropy (1.24) among all of CKII's sites. The average entropy over MEME block 7 and 2 CKII sites is therefore higher than for any other block. MEME block 1 has a wider boxplot than the others, indicating more CKII sites in this block. (B) Average entropy of potential PKC phosphorylation site in the MEME blocks. MEME block 1 and 4 have zero entropy at all their PKC sites. The highest PKC entropy values were observed in MEME block 2 (amino acid 205) and MEME block 7 (amino acid 160) with 1.2 entropy values. MEME block 5, 7 and 11 are unusual in that very few of their PKC sites have zero entropy. MEME block 11 then 7 PKC sites have the highest average entropy. The width of the boxplots indicates that more PKC sites are observed in MEME sites 2, 3 and 7 respectively. (C) Average entropy of potential N-glycosylation site in the MEME blocks. MEME blocks 4 and 5 have zero entropy at all of their ASN sites. MEME block 2, 6 and 9 have nonzero entropy at the majority of their ASN sites. One of the ASN sites (amino acid 99) from MEME block 1 has the highest entropy (1.003) among all ASN sites. The width of the boxplots indicates that more N-glycosylation sites are observed in MEME sites 2 and 7 respectively (D) Average entropy of potential N-myristylation site in the MEME blocks. MEME blocks 1, 2, 4, and 9 have the majority of their myristylation sites possessing zero entropy. The highest myristylation sites entropy is at MEME block 9 and 7 (Amino acid 78 and 160 respectively) with an approximate entropy value of 1.2. MEME block 1 and 7 have more N-myristylation sites than any other block, although MEME block 2 also has a fairly large number of myristylation sites.

CKII sites were detected in MEME blocks 1, 2, 5, 7, 9 and 12; MEME blocks 1, 5 and 9 CKII sites have zero entropy. Unlike other MEME blocks, nearly all of CKII sites at MEME block 2 and 7 have non-zero entropy. One CKII site (position 205-entropy value 1.2) at MEME block 2 is also involved in the co-mutating pair (see below). These results illustrates that despite the high number of potential CKII sites at the highly conserved MEME 1 these sites remain conserved (Figure 5a) and the variable MEME block 2 and 7 undergo amino acid substitutions in CKII sites.

PKC sites were detected in MEME blocks 1, 2, 3, 4, 5, 6, 7, 10 and 11. The conserved MEME blocks 1 and 4 posses PKC sites with zero entropy. The majority of MEME blocks 2 and 3 PKC sites have zero entropy. One amino acid position at MEME blocks 2 and 7 posses the highest entropy of all of PKC's sites. Unsurprisingly, none of the PKC sites at MEME block 11 have zero entropy. The highly variable MEME block 11 has the highest average PKC entropy followed by MEME block 7 (Figure 5b). Four of the PKC sites at MEME block 7 are a part of the co-mutating pairs (see below).

ASN glycosylation sites were detected in MEME blocks 1, 2, 4, 5, 6, 7 and 9. MEME blocks 4 and 5 have zero entropy at all of their ASN sites. MEME block 2, 6 and 9 have nonzero entropy at the majority of their ASN sites. MEME block 1 and 7 are the only blocks with the majority of their glycosylation sites possessing nonzero entropy. Surprisingly, the conserved MEME block 1 also contains the amino acid (position 99) with the highest entropy (Figure 5c); this position is also the amino acid participating in the co-mutation pairs (see below). Additionally, one of the highly variable MEME block 7 N-glycosylation site is also involved in the co-mutation pairs (see below).

Myristylation sites were detected in MEME block 1, 2, 4, 7, and 9. MEME block 1, 2, 4, and 9 have the majority of their myristylation sites possessing zero entropy, in fact all myristylation sites at MEME block 4 have zero entropy, while all but 1 and 2 sites in MEME block 1 and 2, respectively have nonzero entropy (Figure 5d). One of the myristylation sites at MEME block 1, with a relatively high entropy (0.87), is involved in co-mutating pairs (see below).

### Relationship between the high frequency mutation MEME Blocks and previously reported antigenic and receptor-binding sites

MEME blocks 1, 2, 3 and 7 were found to overlap with 4 previously identified antigenic sites (Table 4) [12]. The entire antigenic A site (143–146) was contained within MEME block 7 and overlap a potential phosphorylation site (CKII). The entire antigenic B site (187–196) was contained within one of the repetitive MEME block 2 (179–207) and also contains a potential phosphorylation site (PKC). Notably, antigenic site A also overlaps a hot spot cluster (140–154). As opposed to sites A and B, antigenic sites C and D are represented as single amino acid substitutions. Many of these sites are contained in MEME blocks 1, 2, 3, and 7, with more than 1/5 of the sites in block 2 alone. 43% of antigenic sites in blocks 2 and 80% of antigenic sites in MEME block 3 are also part of a hot spot cluster (200–240). Several of antigenic sites C have a relatively high entropy (over 1), as amino acid position 78 and 205 (data not shown).

**Table 4:** List of antigenic sites observed in the hemagglutinin structure.

**Site**	**Amino Acid Positions**	**Overlaps with...**
**A**	143–146	HA1, MEME7, CKII, ASN
**B**	187–196	HA1, MEME2, PKC
**C & D**	3	MEME10
	31	MEME2
	53	MEME5
	54	MEME5, ASN
	63	MEME5
	78	MEME9, Myristyl
	83	MEME9, CKII
	110	MEME1
	122	MEME1
	133	MEME7
	137	MEME7
	155	MEME7, Myristyl
	164	MEME7
	174	MEME11, PKC
	182	MEME2, ASN
	186	MEME2
	201	MEME2
	205	MEME2, CKII, PKC
	207	MEME2
	208	
	217	MEME3, PKC
	220	MEME3
	226	MEME3
	228	MEME3
	242	MEME3
	260	MEME6
	275	MEME6
	278	MEME6, PKC
	327	MEME4

Antigenic sites A-D [11] were mapped to our consensus sequences and tabulated with overlapping MEME motif, entropy values and post-translational modifications sites. Site A average entropy is based on amino acid position 144 and 145, while site B average entropy is based on amino acid position 188 and 189.

In addition, we correlated the receptor binding sites described by Skehel and Wiley (2000) with MEME blocks. Interestingly, 4 of these receptor binding sites overlap the variable MEME block 7 and the intermediately variable MEME block 2 (Table 5). The receptor binding sites described by Skehel and Wiley (2000) and their overlapping MEME motifs 1, 2, and 7 are presented in Table 5.

**Table 5:** Position of receptor binding sites and their overlap with MEME blocks

**Position of receptor binding sites**	**Overlaps with**
98	MEME1
135	MEME 7
136	MEME 7
137	MEME 7
153	MEME 7
183	MEME 2
190	MEME 2
194	MEME 2

Receptor binding sites described by Skehel and Wiley (2000) were used to generate their correlation with MEME blocks. These receptors binding sites mainly overlap MEME blocks 2 and 7.

**Figure 6:**
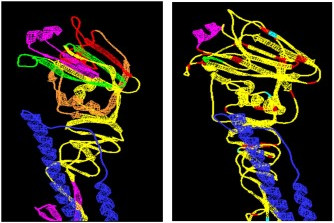
**Graphical representation of MEME blocks and antigenic sites on the 3-D hemagglutinin structure**. The HA1 and HA2 are represented in yellow and blue, respectively. A) MEME blocks on HA: MEME2 (Magenta), MEME7 (Red), MEME3 (Bright Green), MEME1 (Orange (89–129 AA)). B) Antigenic sites on HA: Antigenic Binding Site A (Green), Antigenic Binding Site B (Magenta), Antigenic Binding Site C (Red), Antigenic Binding Site D (Red).

Based on overlapping MEME blocks with hot spots, frequency of amino-acid substitutions, potential post-translational modification sites, receptor-binding sites and antigenic sites we mapped MEME blocks 1, 2, 3 and 7 onto the 3D hemagglutinin structure determined by Fleury and co-workers [13]. Antigenic sites A-D were also mapped for comparison and clarity [11]. Mapping MEME blocks 1, 2, 3 and 7 onto the existing 3-D hemagglutinin structure revealed that these blocks lie on the surface of the protein (Figure 6), specifically on the characteristic 8 beta antiparallel strands of the protein.

### Relationship between co-mutating amino acid pairs and MEME blocks

Co-mutating amino acid pairs were determined based on the best correlating base pairs on a critical value of 95% (r_c _= 0.481894). 107 pairs based on 24 analyzed positions were generated. Of these, 77 pairs contained at least one amino acid within MEME blocks 1, 2, 3 and 7. MEME block 7 contained 66% of these pairs at amino acid position 140-151-153-159-160-161 (Table 6). Interestingly, 4 out of the 6 amino acid positions at MEME block 7 participating in the co-mutating pairs, are potential PKC sites. Additionally, amino acid positions 151 participating in the co-occurring pairs of mutations at MEME block 7 is a potential glycosylation sites. Surprisingly, the highly conserved MEME block 1 participated in co-occurring pairs of mutations in 2 amino acid positions (99 and 363) a glycosylation and a myristylation site, respectively. The highly variable MEME block 11 (171-172-174-176) participated with 4 sites in the co-occurring mutation pairs (Table 6). Interestingly, MEME blocks 3, 4, 5, 8, 10 and 12 had no co-occurring pairs of mutations (Table 6).

**Table 6:** Co-mutating pairs and their position with respect to MEME motifs.

**Co-mutating** **pairs**	**MEME Motif**	**MEME Motif**	**Co-mutating** **pairs**	**MEME Motif**	**MEME Motif**
	**(position 1)**	**(position 2)**		**(position 1)**	**(position 2)**
I-78-D-18	9	**2**	Q-205-I-78	**2**	9
T-99-I-78	**1**	9	Q-205-T-99	**2**	**1**
G-140-D-18	**7**	**2**	Q-205-P-159	**2**	**7**
G-140-I-78	**7**	9	Q-205-T-171	**2**	11
G-151-G-140	**7**	**7**	Q-205-T-176	**2**	11
N-153-D-18	**7**	**2**	S-209-T-99	13	**1**
N-153-I-78	**7**	9	S-209-Q-205	13	**2**
P-159-D-18	**7**	**2**	V-212-N-153	13	**7**
P-159-I-78	**7**	9	V-212-Q-205	13	**2**
P-159-T-99	**7**	**1**	V-260-D-18	6	**2**
P-159-G-140	**7**	**7**	V-260-T-99	6	**1**
P-159-N-153	**7**	**7**	V-260-G-140	6	**7**
G-160-D-18	**7**	**2**	V-260-N-153	6	**7**
G-160-I-78	**7**	9	V-260-P-159	6	**7**
N-161-D-18	**7**	**2**	V-260-G-160	6	**7**
N-161-I-78	**7**	9	V-260-N-161	6	**7**
N-161-G-140	**7**	**7**	V-260-Q-205	6	**2**
N-161-N-153	**7**	**7**	N-264-D-18	6	**2**
N-161-P-159	**7**	**7**	N-264-T-99	6	**1**
T-171-A-14	11	**2**	N-264-N-153	6	**7**
T-171-T-99	11	**1**	N-264-P-159	6	**7**
T-171-P-159	11	**7**	N-264-N-161	6	**7**
K-172-N-153	11	**7**	N-264-Q-205	6	**2**
K-172-P-159	11	**7**	I-294-D-18	-	**2**
K-172-N-161	11	**7**	I-294-T-99	-	**1**
G-174-D-18	11	**2**	I-294-N-153	-	**7**
G-174-G-140	11	**7**	I-294-P-159	-	**7**
G-174-N-153	11	**7**	I-294-N-161	-	**7**
G-174-P-159	11	**7**	I-363-N-153	**1**	**7**
G-174-G-160	11	**7**	I-363-N-161	**1**	**7**
G-174-N-161	11	**7**	I-363-G-174	**1**	11
T-176-D-18	11	**2**	I-363-V-212	**1**	13
T-176-T-99	11	**1**	I-363-I-294	**1**	-
T-176-G-140	11	**7**	V-400-D-18	9	**2**
T-176-N-153	11	**7**	V-400-G-140	9	**7**
T-176-P-159	11	**7**	V-400-N-153	9	**7**
T-176-G-160	11	**7**	V-400-P-159	9	**7**
T-176-N-161	11	**7**	V-400-G-160	9	**7**
			V-400-N-161	9	**7**

77 co-mutating pairs implicated in MEME blocks 1, 2, 3, and 7 were determined using CRASP (see materials and methods) and aligned with MEME motifs.

## Discussion

As opposed to previous molecular and computational approaches to understanding the dynamic nature of the human H3N2 influenza strain, our approach is one of few that attempts to understand and determine the functional importance of variable and conserved motifs in the hemagglutinin protein over time. To the best of our knowledge, this is the first study that addresses different regions in detail, and recognizes novel motifs and identifies their key functional significance with respect to potential post-translational modification sites, co-mutating amino acid pairs, antigenic and receptor binding sites.

In this study we have utilized 17 HA consensus sequences generated from 32 Hong Kong H3N2 isolates spanning the years from 1968 and 1999. We identified 14 MEME blocks, with the clustering of blocks 1, 2, 3 and 7 between positions 85–250 and 430–550 (Figure 6). We correlated the MEME blocks with rates of amino acid substitution and genetic distance. We also utilized entropy plots to determine the clustering of hot spot variability sites. We determined potential post-translational modification sites and correlated their positions and frequencies to MEME blocks, frequency of amino acid substitutions, antigenic sites and receptor binding sites. Out of the 14 MEME blocks, MEME blocks 1, 2 and 3 co-occur more than once within the HA protein and MEME block 7 is a single block. These blocks have different amino acid substitution frequency and encompass different hot spot clusters, post-translational modification sites, antigenic sites and receptor-binding sites. Of these highlighted blocks, MEME 2 had multiple interesting characteristics. This block (29 amino acids) is repeated three times at positions 14–42, 179–207 and 478–506 of the HA protein, and was characterized as an intermediate mutation frequency block (Figure 1). The repetitive nature of this motif could represent multiple binding pockets and could infer specificity to different proteins. Alternatively, such repetitive motif in the HA1 and HA2 subunits suggest common function in the 2 subunits possibly in guiding receptor binding and membrane fusion. A time course analysis to determine the frequency of substitution over the years was performed and lacked a distinct pattern in its amino acid substitution resulting in a zigzag behavior from 1982 onwards (Figure 2c). Additionally, MEME block 2 had one of the highest post-translational modification frequency; having the highest ASN-glycosylation frequency. It was previously reported that the addition of new oligosaccharides to the HA of the H3N2 viruses contributes to the virus ability to elude antibody pressures by changing its antigenic potential [15]. Alterations in HA glycosylation may affect NK cell recognition of influenza virus-infected cells [16]. Additionally, recently circulating avian influenza viruses (H5 and H9 subtypes) mutate at selected N-linked glycosylation sites [14].

MEME block 2 also encompasses the entire length of antigenic site B, and 1/5 of antigenic sites C and D in HA are present in this block (Table 4). Three receptor binding sites overlap this block (Table 5). A high number of co-occurring pairs of mutation was also observed in this block (Table 6). Mutation of glycosylation sites near receptor binding sites of HA1 was proposed to be an adaptation mechanism of the H7 viruses to a new host [18]. These associations suggest that MEME block 2 is a dynamic block in this protein that contributes to the ability of HA1 to mutate, modify its activity by post-translational modification, enhance pathogenicity by mutating receptor binding sites and escaping the host immune response by mutation in antigenic sites.

Additionally, we have identified MEME block 7 (41 amino acids) at position 130–170 (Table 1) as high mutation frequency block (Figure 1). Contrary to MEME 2 block, MEME block 7 revealed a peak frequency of substitution in 1980, corresponding to one of the years with a high mutation rate and therefore this block largely follows the occurrence pattern of substitutions within the entire protein (Figure 2b). However, the overlap between this block and one of the largest hot spots of variability revealed by the entropy plot, namely, the second cluster of hot spots, indicates that increased numbers of mutations within this block is not coincidental (Figure 3). MEME block 7 contained more than 35% of co-mutating pairs (Table 6). This block had the highest post-translational modification frequency (Figure 4), with the highest number of N-myristylation sites (Figure 5b). The entire length of antigenic site A is contained within MEME block 7 (Table 4) and therefore its rapid mutation is a mechanism by the virus to hide from the immune system.

The prevalence of post-translational sites in MEME blocks of high variability, and the lack of conservation observed within post-translational modification sites indicate their importance in sustaining the virus against environmental factors, contribution to viral spread and pathogenicity, and ultimately increasing viral virulence. Increased mutations within ASN-glycosylation sites between our 1968–1999 cohort, is consistent with previous studies, which suggest that increased glycosylation sites attenuates H3N2 viral activity [14]. It was previously reported that the addition of new oligosaccharide to the HA of the H3N2 virus contributes to the virus ability to elude antibody pressure by changing its antigenic potential [15]. Indeed, several studies have addressed the effect of glycosylation on the binding affinity of HA with sialic acid (SA)-containing receptors [16]. A study of reassortment viruses with different H3 HA on naïve mice has shown that, when matched to HK68 NA molecules, viruses with HK68 (7 potential sites) were more virulent than viruses with 12 potential sites (Pan99), and required 3 logs less virus to kill the mice [19]. Another study showed that mutant HA with 3–6 glycosylation sites decreases receptor-binding activity [15]. Additionally, a balance of glycosylation is needed to mediate the interaction of HA and NA molecules for receptor binding activity and viral release [15].

The importance of other post-translational sites that we have observed, such as myristylation sites, is seconded by several studies on the biological role of oligosaccharides and lipid modifications of proteins involved in protein translocation. Myristylation is one of 3 protein lipid modifications which are evolutionarily conserved in plants, animals, and fungi, and which aid in targeting proteins to the plasma membrane and other sub-cellular compartments [16]. Our study of several potential myristylation sites in MEME block 7 and the exhibition of high variability in these sites imply a mechanism by the virus to escape from neutralizing antibodies. On the other hand, the abundance of myristylation sites in the conserved MEME block 1 and their conservation in the years studied, in addition to its overlap with a single receptor binding site infers block 1 importance in selective and specific receptor binding and host cell attachment and infers conservation of essential functions through evolution. Unsurprisingly, minimal post-translational sites were observed in this un-variable block 1. However, the few glycosylation sites observed in MEME block 1 are not conserved and in fact contains the ASN site with the highest entropy and its involvement in the co-mutating pairs suggests specific and selected base pair substitutions over the years. The relatively high co-mutating pairs in this block remain unexplained. Interestingly, the minimal overlap of this conserved site with previously reported antigenic sites [12] is also in agreement with the conserved nature of this site.

The HA protein is on the surface of the influenza particle and is involved in receptor attachment and binding and antigenic determinants. This study reports unique regions in the protein that undergo either high mutation rates to possibly acquire new characteristics by post-translational modification or remain conserved for specific viral functional characteristics. Such changes are expected to enhance the ability of the virus in receptor binding, increase its infective state and escape the host immune response by modification of its antigenic sites. The dynamic evolution of a potentially functional motifs of the HA protein in H3N2 Hong Kong Influenza A virus strains, as revealed by bioinformatics analysis, paves the way for future experimental analysis to determine the significance of these post-translational modification sites and the effect of these alterations on receptor binding and antigenic determinants functions. Because of the worldwide flow in the H3N2 seasonal virus strain and the recently proposed limited mutation of the out of region circulating virus [22] further experimental analysis to appreciate the importance of the variable MEME motif 2 and 7 and the conserved MEME motif 1 in viral pathogenesis is required.

## Methods

### Protein Sequences

Sequence files in the FASTA format were collected from the National Center for Biotechnology (NCBI) Influenza virus resource website [23,24], a specialized database based on the NCBI Genbank database. A total of 34 full-length annotated strains of the HA segment of Hong Kong H3N2 isolates between 1968 and 1999 were used in this study. The utilized HA accession numbers and the equivalent year they were isolated are 1968 [RefSeq:AAK51718, ABQ97200], 1969 [RefSeq:ABB80034], 1971 [RefSeq:ABB82227], 1972 [RefSeqs:ABB80023, ABB04371], 1973 [RefSeqs:ABB04338, ABD60790], 1974 [RefSeqs:ABC40619, ABB04294], 1975 [RefSeq:ABB04928], 1980 [RefSeqs:ABB04283, ABB46547], 1982 [RefSeq:ABB46403], 1983 [RefSeqs:ABB04939, ABB04917, ABB79788], 1984 [RefSeq:ABB79799, ABB04950], 1985 [RefSeq:ABB04327, ABB04305, ABB04349], 1987 [RefSeq:ABB04360], 1988 [RefSeq:ABB04316], 1989 [RefSeq:AAT64734], 1992 [RefSeq:ABB04906], 1999 [RefSeqs:CAC40044, AAK62039, AAK62040, AAK62041, AAK62042, AAK63817, AAK63819, AAK63821]. Using the ClustalW alignment tool [17] a protein consensus sequences was generated for each year as representatives of the entire set of isolates. Seventeen consensus sequences were generated, with an average length of 566 amino acids each.

### Motif and protein modification site prediction in the HA1 protein

Seventeen consensus sequences were submitted into the MEME program [18] to search for motifs that are common to all sequences. A maximum of 50 MEME blocks were generated a motif size varying between 2 and 50 amino acids. Of these, blocks we selected 14 motifs for further analysis that occurred at more than 94% of the sequences.

Consensus sequences were submitted into the PPSearch (Protein Motifs Search) [19] tool available at the European Bioinformatics Institute website. This revealed several post-translation modification sites within the HA1 and HA2 domains. Findings were compared to other motif finding applications including PROSITE under the Expasy Server, PSite, and the ELM database. MEME blocks were also submitted into MAST to determine their functional significance [20]. Additionally, the 1968 consensus sequence was queried against the BLOCKS [21] and PRINTS [21] database to check for the existence of known protein motifs.

### Tabulation of Amino Acid Substitutions and Hot Spots of Variation

Consensus sequences were aligned using the ClustalW multiple alignment tool. Using both the 1968 sequence as a base year, and performing pairwise alignments for each two consecutive years using the LALIGN program of the EMBOSS package [22], the percent change and the number of amino acid substitutions were calculated using the Info align tool. MEGA 4.0 was used to calculate the genetic distances of the HA gene and protein using the Kimura two parameter model and Poisson correction model respectively with gamma-distribution rate across sites [9]. The consensus years that exhibited the greatest frequency of substitutions in the alignment were determined. An entropy plot of the alignment was generated to highlight hot spots of variability. Additionally, conserved regions were determined using BIOEDIT [23], and mapped to MEME blocks and functional motifs.

### Co-occurring pairs of mutations

The ClustalW alignment of the 17 consensus sequence from each year was submitted into CRASP [24] to determine significantly correlated pairs of amino acids which co-mutate within the alignment. Correlating pairs were determined using the Pearson correlation coefficient matrix based on the average accessibility surface area of the amino acids, at a significance level of 95%. The overlap between significantly co-mutating pairs with the MEME blocks was determined and are illustrated in table 6.

## Abbreviations

HA: Hemagglutinin; NA: Neuraminidase;  MEME: Multiple Em for Motif Elucidation; RSCU: Synonymous codon usage; CUP: Codon usage patterns; CKII: Casein kinase II; ASN: asparagines glycosylation; PKC: Protein Kinase C phosphorylation site; Myristyl: myristylation; PPSearch: Protein Motifs Search

## Competing interests

The authors declare that they have no competing interests.

## Authors' contributions

DMAG is a major contributor in the material collection, data analysis and implementation, and writing of the manuscript. MME helped in guiding the study design, implementation and analysis of the data and revised the manuscript. MW helped in the analysis of the correlation between post-translational modification and MEME motifs and generated figures 4 and 5. RS contributed in the initial idea, design and guiding of the project and contributed extensively to the analysis and interpretation of data into the formatted manuscript with extensive revision of the analysis and re-writing of the manuscript to elaborate on its scientific content.

All authors read and approved the final manuscript.
